# Ketamine Sub-Dissociative Dose Vs. Morphine Sulfate for Acute Pain Control in Patients with Isolated Limb Injuries in the Emergency Department: A Randomized, Double-blind, Clinical Trial

**DOI:** 10.30476/BEAT.2021.85949

**Published:** 2021-04

**Authors:** Hooman Esfahani, Zahra Khazaeipour, Arash Safaie, Seyed Mojtaba Aghili

**Affiliations:** 1 *Prehospital and Hospital Emergency Research Center, Tehran University of Medical Sciences, Tehran, Iran*; 2 *Brain and Spinal Cord Injury Research Center, Tehran University of Medical Sciences, Tehran, Iran*; 3 *Prehospital and Hospital Emergency Research Center, Department of Emergency Medicine, Sina Hospital, Tehran University of Medical Sciences, Tehran, Iran*; 4 *Prehospital and Hospital Emergency Research Center, Department of Emergency Medicine, Imam Khomeini Hospital Complex, Tehran University of Medical Sciences, Tehran, Iran*

**Keywords:** Acute pain, Analgesics, Ketamine, Pain management, Trauma

## Abstract

**Objective:**

To compare the ketamine efficacy at a sub-dissociative morphine dose to reduce pain in isolated limb traumatic injuries.

**Methods:**

A double-blind randomized clinical trial study was carried out on patients referred to emergency departments (EDs) due to isolated limb traumatic injuries. Eligible patients were divided into two groups which one group received 0.1 mg/kg ketamine and the other group received 0.05 mg/kg morphine, intravenously. An observed side effect includes pain scores and vital signs were recorded at baseline of every 5 minutes for 30 minutes.

**Results:**

Totally, 73 patients with the mean age of 32.9±10.4 were enrolled of whom 59 (80.8%) individuals were men. The baseline characteristics difference of the two study groups was not statistically significant. The results showed that the change of mean pain score was -6.2 (95% CI: -5.72 to -6.69) points in the group receiving ketamine compared to -5.8 (95%CI: -5.15 to – 6.48) in the group who were administered morphine. At all assessed checkpoints, the pain mean score was lower in the ketamine group than in the morphine group (*p*<0.05); the mean of total pain reduction was greater in the ketamine group during the observation period compared with patients who received morphine (*p*=0.002).

**Conclusion:**

The study findings suggest that the sub-dissociative ketamine efficacy in controlling of the acute pain is not lower than morphine sulfate in patients with isolated limb trauma in ED’s. Thus, it can be considered as a safe and effective alternative approach.

## Introduction

Pain is a common chief complaint among patients referring to the emergency department (ED). The proper pain control is one of the main tasks of any emergency medicine physician (EMP) [1-3]. To the best of our knowledge, there is still no agreement on optimal of analgesic agent for pain reduction in ED’s conscious trauma patients [4, 5]. To alleviate pain, currently, the most prevalent drug used for this purpose in EDs is morphine which is a highly potent opiate that acts directly on pain modulating receptors in the central nervous system (CNS) [6]. It is likely that the standard of morphine dose has few suppressive effects on ED patients with severe pain. Moreover, morphine often has undesirable side effects such as sedation, respiratory depression, hypotension, and nausea [7-10]. Therefore, the finding search for an optimal or alternative agent continues. Ketamine is a phencyclidine-like dissociative agent that has several pharmacologic features, which made it usable and beneficial for pain reduction in EDs. It effectively provides safe analgesia with anxiolysis and no amnesia. In acute care medicine, it is approved for long-term using because it has analgesic properties, serious adverse effects’ low frequency and little effect on the blood pressure and pulse rate in comparison to opioids. Additionally, ketamine use leads to improve analgesia in patients with severe pain (poorly controlled by opioids) and much less opioids administration for such patients. Thanks to the maintenance of pharyngeal reflexes and the airway of unconscious patients, ketamine has kept its position as a favorable tranquilizer in EDs [11-15]. It also has been shown that ketamine shows potent and opioid-sparing effects in sub-dissociative dose and this has centered the drug at the attention heart for analgesia in patients affected by severe pain [11-13]. The current study aimed to compare the ketamine efficacy at a sub-dissociative morphine dose in pain reduction of isolated limb traumatic injuries.

## Materials and Methods

*Study Design*

This study was a single-center, prospective, randomized, double-blind clinical trial performed in a 1000-bed academic teaching hospital affiliated to Tehran University of Medical Sciences (TUMS) in a one-year period from August 2014 to August 2015. The study was approved by the Tehran University of Medical Sciences Ethics and Review Board (Code: IR.TUMS.REC.1394.1710) and was conducted and reported according to the CONSORT. The investigators adhered fully to the principles of the Helsinki declaration throughout the study. The study details were described to participants’ patients by appropriately qualified staff who were completely independent but had sufficient knowledge of this study. Written informed consent was obtained from each patient and ensuring that the patients understood the information. The study was registered at www.irct.ir (IRCT20150213021063N6).

Study Population

Patient’s convenience sample was enrolled during one-year study period. The study were included patients aged 18 to 55 years who presented to the ED for isolated limb traumatic injury with an acute pain score of 5 or more on a standard 11-point (0-10) numerical rating scale (NRS). The excluded patients were those with altered mental status, unstable vital signs, known underlying hepatic or renal diseases and pregnant patients. Enrollment was carried out at various day’ times and week days including weekends. The sample size was calculated as 35 for each arm of the study by considering α=0.05, β=90%, d=0.2, and variance=2. Sample size was estimated by using the following equation: 

n=2z1-α2+z1-β 2σ2d2

Randomization, Blinding, and Intervention

Patient’s enrollment was performed with the first study investigator by completing data sheets. Medications were prepared and delivered to the nursing personnel by the second study investigator in a blinded fashion. All of the research team, patients and an investigator who prepared drug were participated in this double-blinded study. The enrolled patients were divided into the arms of the study by block randomization and were assigned into ketamine or morphine groups. The first group received 0.1 mg/kg ketamine and the second group was administered 0.05 mg/kg morphine in 10 ml normal saline. The first study investigator were recorded the patient’s baseline vital signs and pain score; vital signs and adverse effects were also recorded every 5 and for 30 minutes after drug administration. Additional painkiller (rescue medicine) was administered at any time upon patient’s request or according to the clinical judgment of the in-charge physician during the study. 

Outcome Measurement

The study primary objective was comparing the reductions in patients’ pain based on NRS scores between the two study groups. The secondary goal was to evaluate the need for additional painkiller, side effects, and vital signs changes. 

Statistical Analysis

According to an intention-to-treat principle, all patient’s data was analyzed. A Chi-square test or fisher’s exact test were used to compare rates for categorical measurements and outcome at 30 minutes. ANOVA repeated measure was used to assess differences in pain scores of each group separately over the study period. Furthermore, the independent t-test was performed to evaluate the differences in pain scores between the two groups at different time intervals. All statistical analyses were performed at α<0.05 level and study power was estimated to be 80%. Statistical analyses were conducted by using SPSS version 20 (SPSS Inc. Chicago, III).

## Results

**Fig. 1:**
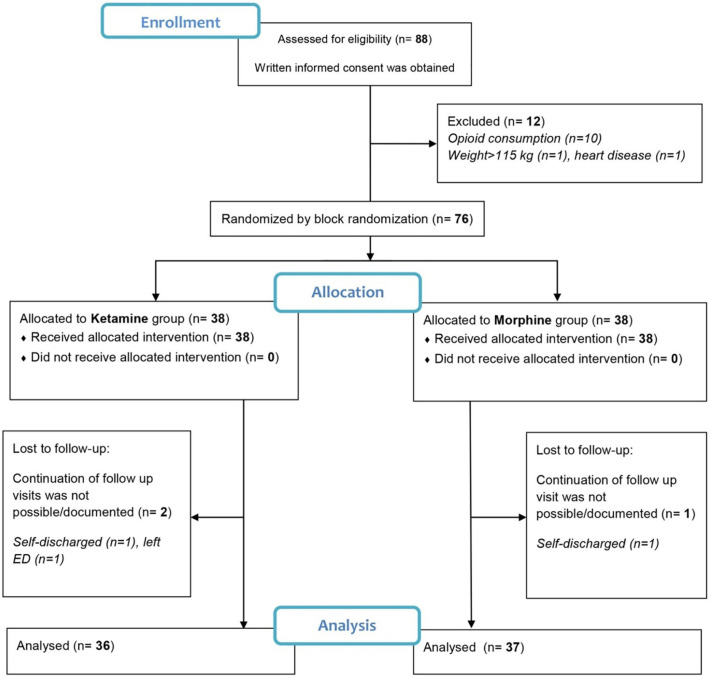
Patient’s flow chart

Patients’ flow chart has shown in Figure 1. In total, 73 patients with the mean age of 32.9±10.4 were enrolled of whom 59 individuals (80.8) were men. The differences in mean age of ketamine and morphine patients’ groups was not statistically significant (32.5±10.0 vs. 33.4±10.8; *p*=0.712). All the study characteristics of participants’ baseline have shown in Table 1. The study findings show that the differences in gender ratio and other measured variables were not statistically significant in the two study groups (*p*>0.05). Table 2 shows the association between baseline pain score, demographic and trauma-related characteristics of the study participants. The terms of transport time was observed to have statistically significant difference (*p*=0.039).

**Table 1:** Comparison of patients’ baseline characteristics in ketamine and morphine groups

**Variable**	**Ketamine (n=36)**	**Morphine (n=37)**	***p*** **-value**
	**Number (%)**		
Gender			0.591^‡^
Male	30 (83.3)	29 (78.4)	
Female	6 (16.7)	8 (21.6)	
Admission type			0.688^‡^
Self- referred to ED	24 (66.7)	23 (62.2)	
Transferred by ambulance	12 (33.3)	14 (37.8)	
Mechanism of trauma			0.524
Car accident	1 (2.8)	1 (2.7)	
Pedestrian	4 (11.1)	4 (10.8)	
Motorcycle	12 (33.3)	9 (24.3)	
Assault	0 (0)	2 (5.4)	
Falling down	6 (16.7)	3 (8.1)	
Sport injury	1 (2.8)	3 (8.1)	
Occupational injury	2 (5.5)	0 (0.0)	
Other	10 (27.8)	15 (40.6)	
Type of injury			0.387
Bone injury	15 (41.7)	20 (54.1)	
Soft tissue	3 (8.3)	6 (16.2)	
Bone and soft tissue	18 (50.0)	11 (29.7)	
Site of injury			0.709
Upper extremity	19 (52.8)	16 (43.2)	
Lower extremity	16 (44.4)	20 (54.1)	
Upper and lower extremities	1 (2.8)	1 (2.7)	
Variable	**Mean±SD**		
Systolic blood pressure (mmHg)	121.7±12.2	119.1±10.4	0.350
Diastolic blood pressure (mmHg)	78.0±6.9	74.8±8.0	0.340
Heart rate (/min)	86.8±8.5	88.2±8.8	0.513
Respiratory rate (/min)	17.3±1.3	17.1±1.1	0.478
Arterial saturation of O_2 _(%)	96.9±1.2	96.6±1.1	0.300
Baseline mean pain score (NRS)	8.4±1.5	8.9±1.3	0.130

^‡^Based-on Pearson Chi-Square test

**Table 2:** Association between baseline pain score and demographic and trauma-related characteristics of study participants

**Variable**	**Mean score of pain at baseline (SD)** ^a^	***p*** **-value**
Age groups		0.318
18-25	8.9 (1.1)	
26-40	8.5 (1.5)	
41-55	8.6 (1.6)	
Gender		0.318
Male	8.6 (1.5)	
Female	8.9 (1.9)	
Admission		0.039
Self-referred to ED	8.3 (1.5)	
Transferred by ambulance	9.1 (1.1)	
Type of injury		0.783
Bone injury	8.8 (1.6)	
Soft tissue injury	8.6 (1.7)	
Bone and soft tissue injury	9.1 (1.2)	
Site of injury		0.382
Upper extremity	8.5 (1.5)	
Lower extremity	8.8 (1.2)	

SD: Standard deviation; a. Based on NRS

Main Outcomes: Pain Reduction 

**Fig. 2:**
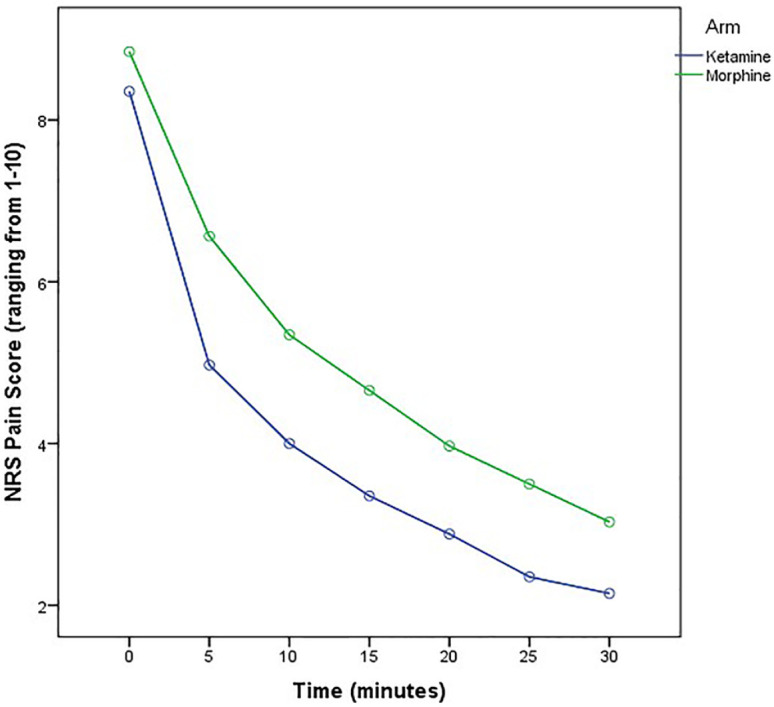
Pain mean score over the time by treatment group

**Fig. 3:**
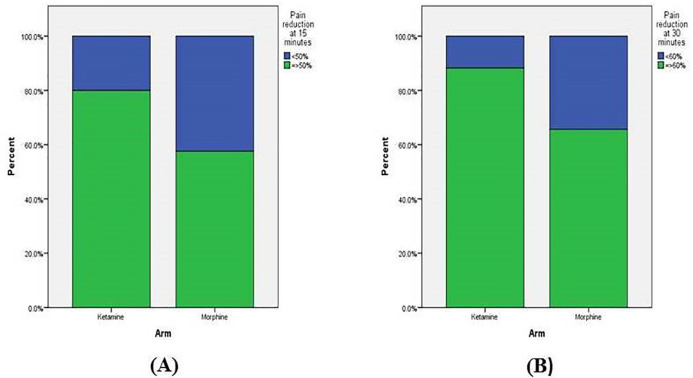
**(A)** Achieving patients’ percentage more than 50% pain reduction in the first 15 minutes; (**B**) Achieving patients’ percentage more than 60% pain reduction in the first 30 minutes

A downward trend was observed in pain mean score of both study group. The results showed that the change of pain mean score was -6.2 (95% CI: -5.72 to -6.69) points in the group receiving ketamine compared to -5.8 (95% CI: -5.15 to – 6.48) in the group who were administered morphine. As it is depicted, pain mean score was constantly decreased over the study period in both groups. However, the mean score of pain was lower in the ketamine group than in the morphine group, and pain reduction was greater in the ketamine group compared with patients who received morphine (*p*=0.002) at each measurement time (Figure 2). The patient’s percentage who have reached a 50% pain reduction level is a practical index, which has been used to assess the pain reliever’s efficacy. As it shows in Figure 2, around 80% of patients’ ketamine group showed more than 50% pain reduction in the first 15 minutes while only 60% of the morphine group has reached this level (*p*=0.066). Additionally, patients with a higher statistically percentage were reached 60% pain reduction in the ketamine group compared with the morphine group (*p*=0.040) (Figure 3). Only 2 patients (5.6%) received rescue analgesia in ketamine group compared with those in morphine groups who need rescue analgesia and was around 2-fold higher (13.5%). However, the study shows that there is no statistically significant difference between the two groups (*p*=0.430).

Side Effects I: Vital Signs During Pain Reduction Treatment 

Changes includes systolic and diastolic blood pressure, heart rate, respiratory rate and arterial oxygen saturation were also monitored and compared between the two groups in the study. There was no statistically significant differences between the two groups (Table 3).

**Table 3:** Pain mean scores over time by treatment groups

**Time**	**Ketamine group**	**Morphine group**	***p-*** **value**
	**Mean score of pain (SD)** ^ a^		
Baseline	8.4 (1.5)	8.9 (1.3)	0.002
5 min	5.0 (2.8)	6.6 (2.0)	
10 min	4.0 (2.2)	5.3 (1.6)	
15 min	3.4 (1.6)	4.7 (1.7)	
20 min	2.9 (1.5)	4.0 (1.3)	
25 min	2.4 (1.2)	3.5 (1.2)	
30 min	2.1 (1.2)	3.0 (1.3)	

SD: Standard deviation; a. Based on NRS

Side Effects II: Complications Due to Pain Reduction Treatment 

Due to the pain reduction treatment, complications were another investigated outcome in the current study (Table 4). The most prevalent side effect was dizziness in both ketamine and morphine groups which has reported as a lightheadedness. It was quite high in the ketamine group; however, we found no statistical difference between the two study groups. Finally, the overall side effects of percentage were compared in the two groups. The results showed that there is a higher percentage in the ketamine group than in the morphine group and the difference was statistically significant (*p*=0.009). No noticeable adverse effect was reported necessary of the study withdrawal.

**Table 4:** Observed side effects in the two study groups

**Side effect **	**Ketamine (n=36)**	**Morphine (n=37)**	***p*** **-value**
	**Number (%)**		
Dizziness	10 (27.8%)	7 (18.9%)	0.417
Nausea	5 (13.9%)	1 (2.7%)	0.107
Vomiting	1 (2.8%)	0 (0.0)	0.493
Hypotension	2 (5.6%)	0 (0.0)	0.240
Tachycardia	1 (2.8%)	1 (2.7%)	1.000
Disorientation	1 (2.8%)	0 (0.0)	0.493
Total	20 (55.6%)	9 (24.3%)	0.009

## Discussion

The pain management is a great importance in trauma patients referring to EDs. For a long time, opioids use has been an acceptable option to alleviate pain. The current study compared the morphine and ketamine effects of tranquilizing pain in trauma patients. The results of the study indicated that sub-dissociative ketamine doses is more effective than low morphine doses in pacifying pain. This asset enables pain management with minimum side effects by requiring no medical intervention or intensive care for patients. The current study results were in agreement with the study of Motov *et al*., [16] which showed that there is no significant differences in the patients’ pain relief who were treated with either ketamine or intravenous morphine and the study suggested that ketamine is appropriate for using in emergency departments with the same efficacy [16]. Majidinejad *et al*., [17] also reported that ketamine and morphine have equal pain reduction which is in contradiction with the current findings. In the current study, pain reduction was significantly faster in the ketamine group than in the morphine group. According to the current results, approximately 80% of the study patients achieved 50% pain reduction 15 minutes after drug injection in the ketamine group while this rate was almost 60% in morphine group. This findings was supported by previously published research carried out by Miller *et al*., [18] who reported slower pain reduction with morphine. To reduce pain, the fastest response was identified in the first 5-minutes interval of a 30-minutes time while the fastest response in the study of Motov *et al*., [16] was observed 15-30 minutes after beginning of drug administration. In these studies, this discrepancy may be due to the use of ketamine and morphine doses. Similar results were obtained by Miller *et al*., [18] who compared the effectiveness of a ketamine sub-dissociative dose vs. intravenous morphine to reduce pain in patients referred to the ED. They indicated that 0.3 mg/kg ketamine dose did not indicate a superior maximum reduction in numeric rating pain scores scale when compared to 0.1 mg/kg morphine dose. Notably, none of the received ketamine patients showed serious adverse effects that is compatible with the Lester *et al*., [19] and Richards *et al*., [11] studies. These studies reported high safety and low adverse effects for ketamine in pain relief. Miller *et al*., [18] and the current study reported no major adverse effect for low ketamine dose. In addition to the above statements, some studies suggested that ketamine to opioids supplement can boost the pain relief potency in patients and post-operative situations that urge physicians to employ a combination therapy for patients who suffer from acute pain. Overall, there is no agreement on the ketamine dosage for these patients who refer to the ED. It seems that further studies need to possess a large patients’ cohort and it will increase our understanding of how much ketamine is needed to manage pain with respect to the numeric rating scale.

In conclusion, the study findings suggest that sub-dissociative ketamine can be consider as a safe and effective approach to control acute pain in trauma patients with isolated limb refer to EDs. It also improves pain relief significantly when compared with intravenous morphine. Notably, ketamine is associated with minor side-effects which are clinically negligible. The ketamine palliative effect has a more rapid onset when compared with morphine.

## Limitations

The small sample size is a major limitation of the current study which could affect the findings. The current study was also carried out at a single site which could generate a heterogeneous sample regarding medical emergency conditions and, consequently was reduced the results’ generalizability. Patients were followed for only 30 minutes while this following could provide more and different information for 1 or 2 hours by assessing a long-term pain killers effects. 

## Ethical Approval:

Ethical approval was obtained from the Tehran University of Medical Sciences Ethics and Review Board (No. IR.TUMS.REC.1394.1710).
